# Sudden Unexpected Postnatal Collapse: A Retrospective Cohort Study Describing Incidence, Risk Factors and Role of Perinatal Transition

**DOI:** 10.1111/apa.70670

**Published:** 2026-07-13

**Authors:** David Forsberg, Louise Steinhoff, Gustaf Drevin, Helena Idborg, Nicolas J. Pejovic, Eric Herlenius

**Affiliations:** ^1^ Department of Women's and Children's Health Karolinska Institutet Stockholm Sweden; ^2^ Astrid Lindgren Children's Hospital Karolinska University Hospital Stockholm Sweden; ^3^ Center for Molecular Medicine, L8:05 Karolinska Institutet Stockholm Sweden; ^4^ National Board of Forensic Medicine Stockholm Sweden; ^5^ Department of Medicine Solna Karolinska Institutet Stockholm Sweden; ^6^ Department of Global Health Karolinska Institutet Stockholm Sweden

**Keywords:** perinatal transition, postnatal collapse, prostaglandin, skin‐to‐skin, sudden infant death syndrome

## Abstract

**Aim:**

This study aimed to investigate the incidence, risk factors and possible aetiology of sudden unexpected postnatal collapse (SUPC), a potentially fatal yet poorly understood event.

**Methods:**

In a retrospective cohort, patient records from 483 284 infants born in Stockholm, Sweden, between 2002 and 2022 were screened for SUPC‐related diagnoses. SUPC was defined as a collapse within the first 7 days of life in near‐term infants with good postnatal adaptation. Maternal, infant and event characteristics were collected. Urine was analysed for prostaglandin E_2_ (PGE_2_) metabolites in a subgroup of cases.

**Results:**

We identified 149 cases of SUPC, corresponding to an incidence of 31 per 100 000 live births, exceeding that of early onset sepsis caused by Group B Streptococcus. Half the SUPC cases occurred during the first four postnatal hours. The mortality was 7% and 26% suffered from neurological sequelae. The highest risk of SUPC coincided with the postnatal peak in PGE_2_ levels.

**Conclusion:**

All newborn infants were at risk of SUPC, especially within 24 h of birth. SUPC was associated with early unsupervised skin‐to‐skin care and bed sharing. Guidelines promoting *safe* sleep and skin‐to‐skin care should be implemented to prevent SUPC and save the lives of newborn babies.

## Summary

Sudden unexpected postnatal collapse (SUPC) is a potentially catastrophic yet largely under‐recognized event. SUPC is more common and has a higher mortality than sepsis caused by Group B Streptococcus, and there is a connection between SUPC and skin‐to‐skin care especially immediately after birth, when prostaglandin E_2_ levels are high. Most SUPC could likely be prevented by improvement of existing guidelines on infant care during the first days of life.

## Graphical abstract

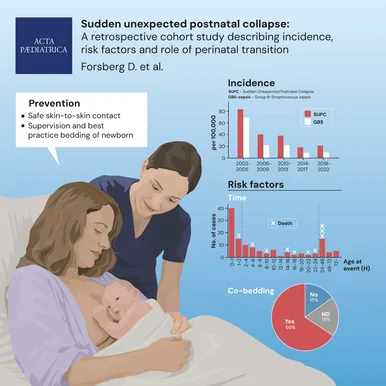

**Abbreviations**
ASD: atrial septal defect
BE: base excess
BRUE: brief resolved unexpected event
CCHS: congenital central hypoventilation syndrome
EEG: electroencephalogram
GBS: Group B Streptococcus
HIE: hypoxic ischaemic encephalopathy
ICD‐10: international classification of disease, 10th revision
PGE_2_
: prostaglandin E_2_
SIDS: sudden infant death syndrome
SUDI: sudden unexpected death of infancy
SUPC: sudden unexpected postnatal collapse

## 1 Introduction

Sudden unexpected postnatal collapse (SUPC) is a potentially deadly or neurologically catastrophic event that occurs in seemingly healthy newborn infants [1]. However, there is no clear consensus on terminology, definitions or criteria. Moreover, cases overlap with conditions diagnosed as apparent life‐threatening event, brief resolved unexpected event (BRUE), sudden unexpected early neonatal death, sudden unexpected death in infancy (SUDI) and sudden infant death syndrome (SIDS) [2, 3, 4, 5].

Furthermore, no International Classification of Diseases, 10th revision (ICD‐10) code exists for SUPC at present. This limits the comparability of studies performed. Overlapping terminology and a large variation of inclusion criteria has resulted in a widespread incidence ranging from 2.6 to 133.0 cases per 100 000 live births 1977–2022 [2, 6, 7]. Another limitation to the present knowledge of SUPC is that most publications were case reports only including a few patients, and the data often relied on reports by either parents or hospital staff [2]. The aetiology of these conditions remains unknown. However, several risk factors have been proposed. These have included a primiparous mother, prone position, early and unsafe skin‐to‐skin care and co‐bedding [2, 8, 9]. The triple risk model proposed in the diagnosis of SIDS with three concurrent factors (vulnerable infancy and a genetic or underlying predisposition in combination with an exogenous stressor) may also explain SUPC‐like conditions [10, 11]. Moreover, dysfunctions in the brainstem control of respiration have been suggested as an underlying factor in cardiorespiratory problems in infants. This was related to infection and could be mediated by the actions of prostaglandin E_2_ (PGE_2_) [12, 13] Notably, levels of PGE_2_ were also increased immediately after birth [14].

The aim of this study was to establish the incidence of SUPC, based on a larger population over a longer period of time. We applied distinct inclusion criteria and clearly defined the condition and provided information on risk factors and outcome. All seven delivery wards in the catchment area of Stockholm County, Sweden, were included establishing a regional cohort study of close to 500 000 live births.

## 2 Method

We retrospectively investigated SUPC in seemingly healthy newborn infants born at ≥ 35 weeks of gestation during a 21‐year period (2002–2022) at seven major delivery wards and neonatal centres in Stockholm County, Sweden. The included hospitals were Karolinska University Hospital Solna (2002–2022), Karolinska University Hospital Huddinge (2006–2022), Danderyd Hospital (2006–2022), BB Stockholm (2008–2022), Södersjukhuset/Sachs' Children and Youth Hospital (2007–2022), BB Södertälje (2007–2022), and BB Sophiahemmet (2015). The specific time periods were selected based on when the respective hospitals progressed to using electronic health records. Only electronic health record data were screened in this study. BB Sophiahemmet was only active during 2015. These centres accounted for about 30 000 births per year corresponding to a quarter of all births in Sweden.

The study cohort was selected from all in‐hospital live births during the defined time period in the geographical location. As no code for SUPC exists in the ICD‐10, we actively looked for SUPC cases by searching for diagnoses suggestive of or potentially relating to a collapse (Table 1). The search was made in the antenatal, perinatal, and neonatal chart registries Obstetrix (Siemens, Munich, Germany) and TakeCare (Compu Group Medical, Arizona, USA). We also added a search for diagnoses concerning SIDS and other unexpected infant deaths to identify SUPC cases leading to death. Premature infants born before 35 weeks of gestation or infants with an umbilical artery pH < 7.0 or base excess >− 12 at birth were excluded. Among the remaining infants, the electronic health records were systematically investigated to characterize the circumstances around the collapse. The inclusion criteria were SUPC within 7 days after birth with documentation of cyanosis, apnoea and loss of tone and the requirement of resuscitation (ventilation using bag and mask or Neopuff). This is in accordance with established international guidelines and definitions from the British Association of Perinatal Medicine [15, 16, 17, 18, 19].

**TABLE 1:** Diagnoses included in the screening for sudden unexpected postnatal collapse cases.

ICD‐10	Diagnosis
P21.0	Severe asphyxia at birth
P21.9	Asphyxia at birth
P28.2	Cyanotic attack of newborn
P91.6A‐C	Hypoxic‐ischaemic encephalopathy (HIE) I‐III
P28.3	Primary sleep apnoea of newborn
P28.4	Other apnoea of newborn
G47.3	Sleep apnoea syndrome
I461	Sudden cardiac death
R68.13	Apparent life‐threatening event in infant (ALTE)
R95.0	Sudden infant death syndrome with mention of post‐mortem
R95.9	Sudden infant death syndrome without mention of post‐mortem
R96.0	Instantaneous death
R96.1	Death occurring < 24 h from onset of symptoms, not otherwise explained
R99.9	Unspecified cause of death

On account of being newborn infants, patients were not directly involved in the study.

Infants were identified using personal identification numbers. Medical data, including examinations and diagnostic and therapeutic data, were collected from the electronic health records and further investigated to determine inclusion or exclusion in the study on a case‐by‐case basis. These included follow‐up notes and interviews with staff involved in the initial event. Patient follow‐up after discharge was not evaluated in this study.

Swedish law mandates that any death which may be unnatural in manner should be subject to police involvement and forensic medical examination. Full post‐mortem reports for infants that died in relation to a SUPC event were obtained from the National Board of Forensic Medicine in Sweden. Records were obtained under the Swedish law of principle of public access to official records by a governmental body.

Patients not fulfilling the inclusion criteria and patients with any known plausible cause were excluded (Table S1). Thus, the study only included seemingly healthy newborn infants with an unexpected collapse requiring resuscitation (Figure 1). For each case, we identified characteristics with regards to pregnancy and birth (Table S2). To minimize bias, we rigorously examined suspected SUPC cases through two standardized protocols and applied easily identifiable inclusion and exclusion criteria.

**FIGURE 1:**
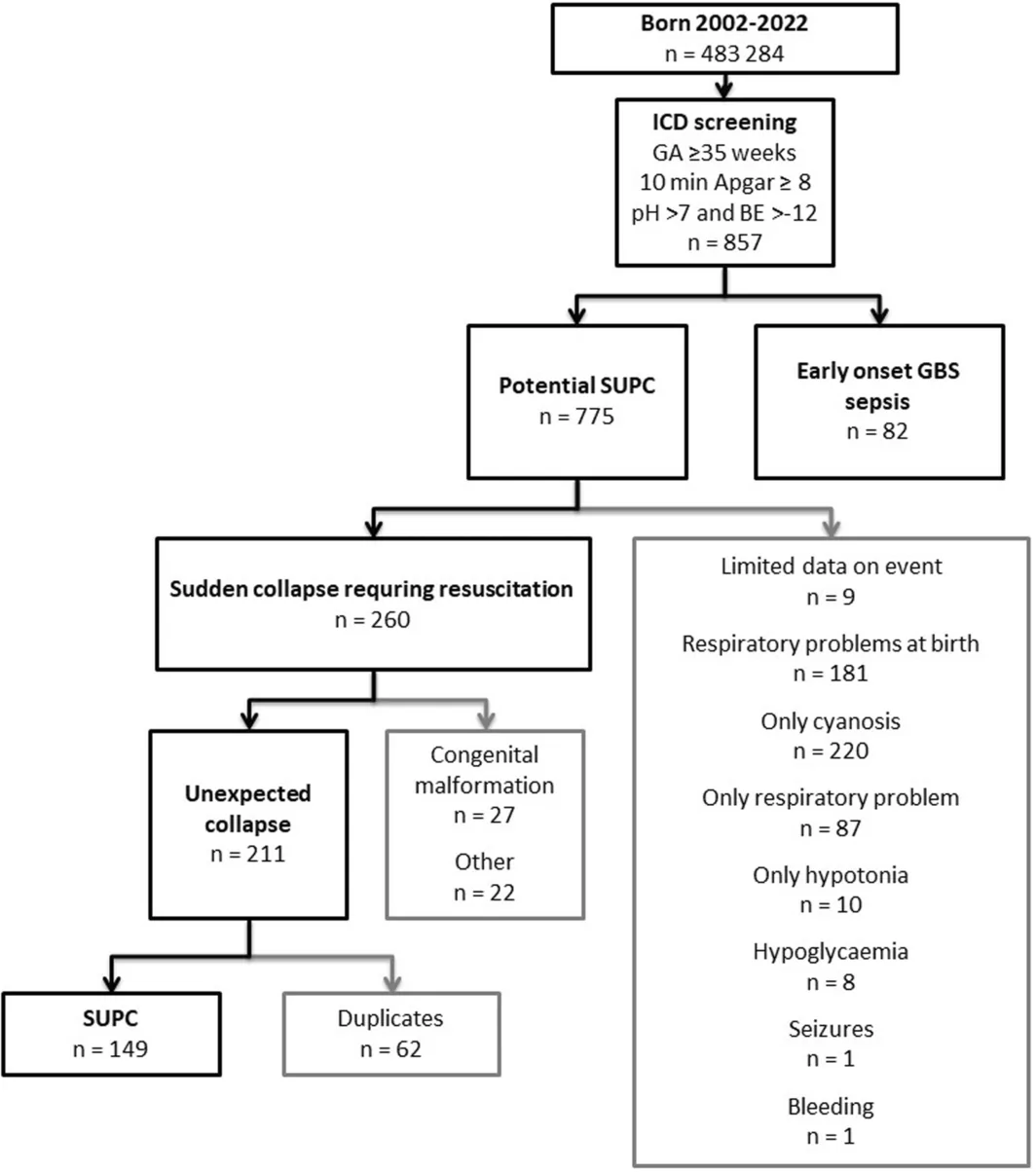
Inclusion flow chart. Of the 483 284 infants born alive during the study period, we identified 857 potential cases fulfilling criteria for non‐complicated birth at full‐term based on ICD‐10 diagnoses. Among these, 149 remained after reviewing electronic health records for inclusion and exclusion criteria. In addition, 82 infants were diagnosed with early‐onset Group B streptococcus (GBS) sepsis.

Infants from a previous study were included as the study periods and regions overlapped (1), and for these infants additional electronic health record data were added for the present study.

Since 2011, we have conducted a prospective study, where new SUPC cases were included. Blood and urine samples were collected from these infants and a subset was analysed for PGE_2_ metabolites, with the same method as described previously [20]. Controls were recruited from Karolinska University Hospital prospectively and then age‐matched to the SUPC cases. The controls all had uncomplicated deliveries and no need for any additional interventions during the first week of life.

For incidence comparison, we investigated patients with a diagnosis of early onset Group B streptococcus (GBS) sepsis (ICD‐10 code P36.0) from the same cohort. Inclusion criteria were the presence of blood or cerebrospinal fluid culture positive for GBS and treatment with antibiotics. Exclusion criteria were the same as for SUPC. Premature infants born before 35 weeks of gestation or infants with an umbilical artery pH < 7.0 or base excess >− 12 at birth were excluded. Thus, we only included GBS sepsis in apparently healthy near‐full term infants. GBS sepsis is a well‐known disease for which efforts have been made to reduce incidence and improve outcome over the last decades [21]. To compare pregnancy and birth data, we used the Swedish National statistics on pregnancy, birth and newborn infants [22].

### 2.1 Statistical Analysis

For each comparison, we performed a multivariate analysis of variance. Missing data were either excluded from the analysis or included as missing data (categorical variable). All calculations for the statistical tests were conducted with JMP PRO 18.0 (SAS Institute Inc., Cary, North Carolina, USA). In all cases, *p* < 0.05 was considered statistically significant.

### 2.2 Ethics

Ethical permission to screen the birth registry was obtained from the Swedish Ethical Review Authorities (ID 2011/1891, 2011/1908‐31, 2011/1908‐32, 2017/1385‐32 and 2022‐02748‐02) and all patient data were anonymized.

## 3 Results

We identified 149 cases of SUPC in seemingly healthy infants among the 483 284 live born infants (Figure 1). Thus, the incidence was 31 per 100 000 live births, which was higher than previous investigations of SUPC [1, 2, 8, 18, 23]. However, the incidence did decline during the study period (Figure 2a). This is possibly related to increased awareness and establishment of national guidelines for newborn care [9, 15, 17]. Among the 483 284 live born infants, 16 785 (3.5%) were excluded due to prematurity (gestational age < 35 weeks).

**FIGURE 2:**
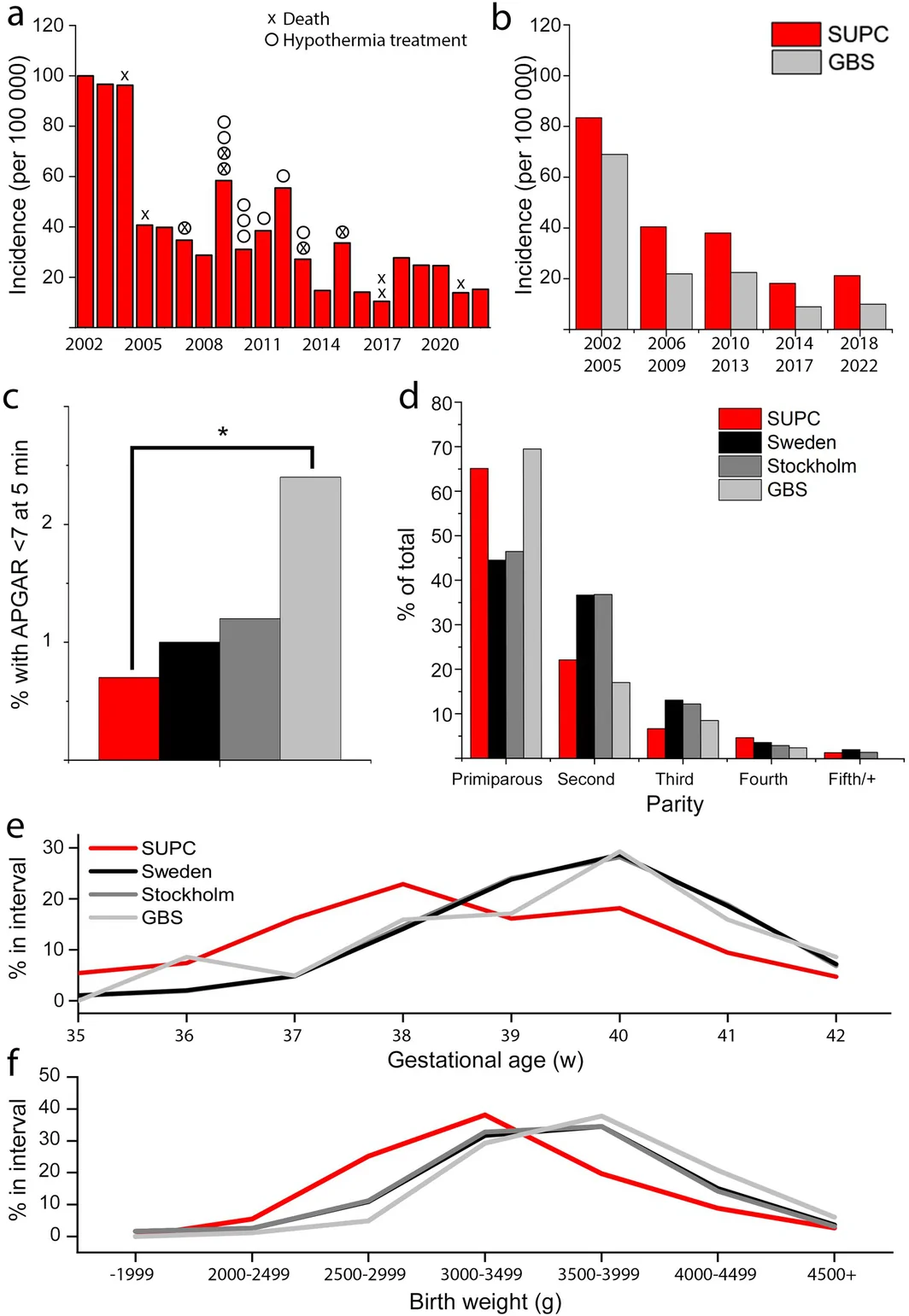
Incidence and birth characteristics. The incidence of sudden unexpected postnatal collapse (SUPC) has declined throughout the study period (a). However, the incidence of SUPC was higher than that of early onset group B streptococci (GBS) sepsis (b). Fewer SUPC cases had low APGAR scores at 5 min than infants with GBS sepsis (c). Among the SUPC cases, most mothers were primiparous (d). SUPC cases displayed a significantly lower gestational age (e) and birth weight (f) compared to national (Sweden) and regional (Stockholm) newborn infants, as well as the included infants with GBS. Data between 2002 and 2005 only includes Karolinska University Hospital Solna. *p < 0.05.

Additionally, we found 82 cases of infants diagnosed with early onset GBS sepsis (Figure 1). Equivalent to an incidence of 17 per 100 000 live births. Thus, the incidence of SUPC is higher than that of early onset GBS sepsis (Figure 2b).

The average Apgar scores were 8.1 ± 1.6 at 1 min, 9.5 ± 0.9 at 5 min and 9.8 ± 0.5 at 10 min. Among the SUPC cases 0.7% had an Apgar score of < 7 at 5 min. Thus, almost all included cases exhibited a good immediate postnatal adaptation. This was similar to the regional (Stockholm County) and national (Sweden) data (1.0% and 1.2% respectively). Fewer SUPC cases had a low APGAR score compared to infants diagnosed with early onset GBS sepsis (2.4%, *p* < 0.05) (Figure 2c). Umbilical artery pH at birth was 7.24 ± 0.09, which was comparable to healthy babies in the same region (7.25 ± 0.07) [24]. We therefore concluded that SUPC were not related to adversities around birth. SUPC cases showed a higher proportion of primiparous mothers compared to both regional and national data (64% vs. 47% and 45%, *p* < 0.05) (Figure 2d). No difference in mode of delivery (vaginal or caesarean section) was observed. However, SUPC cases were born at a slightly earlier gestational age (*p* < 0.05) and had a lower birth weight (p < 0.05) compared to the regional and national population (Figure 2e,f).

The majority of SUPC cases (81%) occurred within 24 h of birth and half (50%) within the first 4 h (Figure 3). We could not see any relation to time of day. This was in contrast to Poets et al. [25] who showed that most cases occurred during the night or morning. However, these data were not available in most previous case‐reports nor cohort studies [8]. Two‐thirds, 96 (66%), of the SUPC cases were associated with bed sharing (sleeping in the same bed as the caretaker), similar to SIDS [26]. This was in accordance with several previous studies [2, 9, 27]. Many cases, 46 (31%), lacked data on whether the parent was asleep during the collapse. However, almost all cases, six (86%), when the parent was known to be asleep, ended in death of the infant. We also found that six (60%) cases ending in death occurred during skin‐to‐skin care, and 60 (40%) cases occurred during skin‐to‐skin care with the baby on the parent's chest (Table 2). In 31 (21%) cases there was evidence of an asphyxia related position where the infant's face was covered at the time of the collapse. However, no data on head position were available in 88 (59%) cases. In 61 (41%) cases there were insufficient data to determine if the infant had been in an asphyxia related position. This raises doubts on whether the skin‐to‐skin care taking place was actually safe. Thus, skin‐to‐skin care, while beneficial, needs to be implemented with caution [9, 16, 27] and requires sufficient parental awareness and observation by health care staff.

**FIGURE 3:**
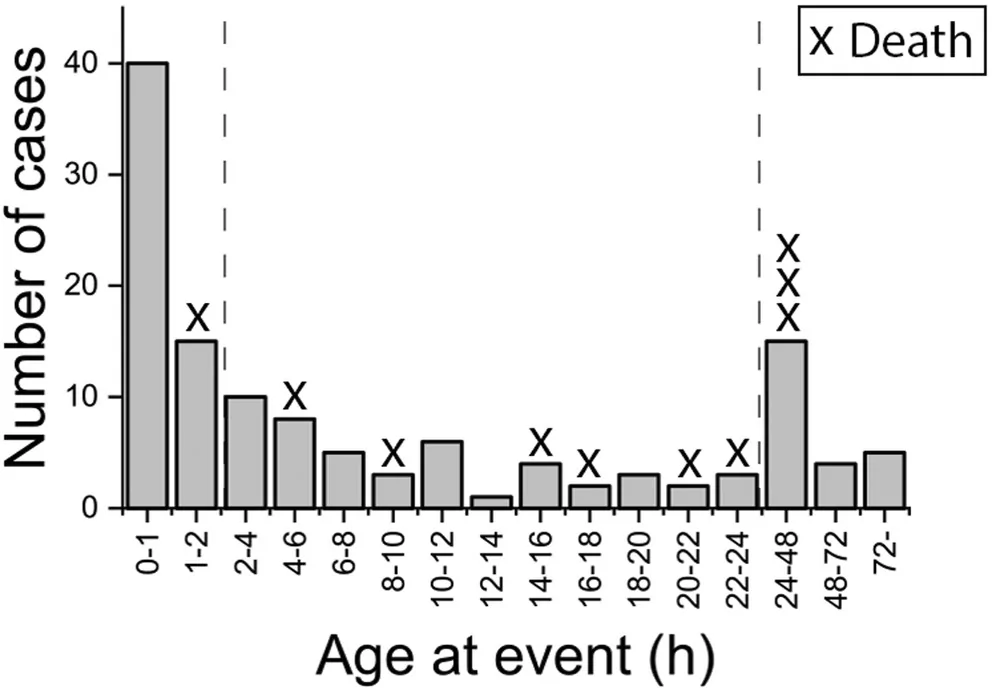
The first 24 h are important. Half (52%) of the SUPC cases occurred within 4 h after birth. An additional third (82%) occurred during the first 24 h. In the figure, the Age at event scale is different between 0–2 h, 2–24 h and 24 h, marked by dashed lines.

**TABLE 2:** Location the infant was found.

Place found at time of collapse	*n* (%)	Face covered	*n* (%)
Parent's chest	60 (40)	Yes	13 (22)
		No	10 (17)
		No data	37 (61)
With parent	29 (20)	Yes	15 (52)
		No	12 (41)
		No data	2 (7)
In bed (with parent)	10 (7)	Yes	3 (30)
		No	5 (50)
		No data	2 (20)
In bed (alone)	22 (14)		
Other^a^	3 (2)		
No data available	25 (17)		

^a^ With hospital staff (*n* = 2) or while being cleaned (*n* = 1).

A relation to breastfeeding (collapsing during or just after breastfeeding) was found in 62 (42%) cases. The age of the infant at the time of collapse explains why most events occurred either in the delivery room, 60 events (40%), or the maternity ward, 68 events (45%). A majority, 129 (87%), of cases collapsed while in the care of the parents. Only nine (6%) of the included SUPC cases collapsed at home. In most cases, 105 (70%), the SUPC event gave no sustained (> 24 h) symptoms. However, 39 (26%) infants exhibited symptoms of neurological damage (Table 3). Among these, one fifth developed hypoxic ischaemic encephalopathy grade I (HIE I) and one in four developed HIE II. Two sudden unexpected postnatal collapse cases resulted in a HIE III. Other neurological symptoms (Table 3) included seizures, hypotonia and epileptic activity on electroencephalogram (EEG) examination. In previous studies, surviving infants have been shown to suffer from neurological sequelae at discharge in 17%–60% [2, 8, 25]. In our study 13 patients were treated with hypothermia after the collapse. Of these, all but one developed neurological symptoms or HIE. The outcome of SUPC‐related conditions is poorly studied. In the most catastrophic cases they result in the death of the infant (SUDI, SUEND, SIDS). Between 27% and 41% have been reported to die following the event [8, 25]. We identified 10 deaths following SUPC. The mortality (7%) was lower compared to previous studies. Notably, we excluded cases where early onset GBS sepsis was known prior to the collapse, which has been deemed an important cause of death [8]. The number of SUPC cases that died each year remained constant throughout the study period, despite the decrease in overall incidence.

**TABLE 3:** Initial outcome of SUPC cases.

Outcome	*n* (%)		*n* (%); *n* dead (%)
No adverse outcome	105 (70)		
Neurological symptoms	39 (26)	HIE I	8 (20)
		HIE II	8 (20); 1 (3)
		HIE III	2 (5); 1 (3)
		Other^a^	22 (46); 8 (21)
Death	10 (7)		

^a^ Seizures, epileptic activity on EEG, prolonged/sustained hypotonia, spasticity and symptoms of meningoencephalitis.

Of the 10 SUPC cases that died, seven collapsed within 24 h of birth. This is in contrast to [18], in which only 11% of collapses resulting in death occurred within 24 h. All of the infants who died were found collapsed during co‐bedding and half (50%) of the dead infants collapsed at home. Forensic post‐mortem was performed in nine of the 10 infants. The manner of death was found to be natural in six of the cases (Table S3), while three were deemed accidental and one was undetermined. Only one case was diagnosed and classified as SIDS with no findings of any significance at post‐mortem (Table S3). Two cases had congenital heart malformations, precluding a diagnosis of SIDS. One infant was found to have a metabolic disease. One case was diagnosed as severe lung disease. The infant who did not undergo post‐mortem was deemed to have died from bilateral spontaneous pneumothorax. The causes of death identified in our study were similar to those found in previous studies regarding SIDS [8].

In total, four of the post‐mortem cases showed a cause of death related to airway obstruction or hypoxia in the form of hypoxic brain injury, organ failure and positional asphyxia. The manner of death was deemed accidental in three of these, with two cases showing factors associated with co‐bedding. The third case of accidental asphyxiation was diagnosed as positional asphyxiation and had no findings at post‐mortem but a convincing history of co‐bedding and death scene investigation indicating an asphyxiated position. The fourth case, which was deemed to be secondary to respiratory and cardiac arrest of unknown aetiology, was considered undetermined.

Following collapse, 48 (32%) infants were investigated with brain ultrasound, 54 (36%) with EEG and 56 (41%) with echocardiography. However, in only 63 (42%) cases a blood gas analysis was performed and in 42 (28%) this was sampled within 12 h of the collapse. We could observe a declining trend over the years in the use of EEG and echocardiography following SUPC. Conversely, there was an increase in blood sampling, especially blood gas analysis, after the event over time. Acute blood gas analysis may detect mild cases where specialized investigations might not be necessary, explaining the decline in these types of investigations (Figure S1).

The post‐event investigations discovered a plausible risk factor or cause of the SUPC in only 20 (13%) cases (Table 4). Among these, congenital heart malformations, including atrial septal defects, patent ductus arteriosus, patent foramen ovale and SCN5A‐mutation, were the most common, identified in seven (6%) cases. Thus, SUPC could be the initial presenting of several diseases and malformations (Table 4) in newborn infants. Of the events with an unknown cause, 86 (67%) occurred during bed sharing. As PGE_2_ levels were known to be increased following birth [28] and directly affect respiratory control [12, 13, 29], we set out to investigate the possible connection between PGE_2_ and SUPC. In the study, urine samples were obtained in 10 of the SUPC cases and in an additional 14 age‐matched control infants. Levels of urinary PGE_2_ metabolite were higher in SUPC cases the first 48 h (*p* < 0.05). In both SUPC cases and control the levels significantly decreased over the first days of life (*p* < 0.0001). This was in accordance with previous studies showing increased levels of prostaglandins in umbilical artery samples before delivery and immediately after birth [28]. The urinary PGE_2_ metabolite levels remained elevated the first 3 days postnatally in all examined infants (Figure 4). This is the time‐window when most SUPC events occurred. The high levels of PGE_2_ have been shown to be important for the transition to extra uterine life [30], promoting deep breaths [29, 31] and relaxation of the airways [32]. PGE_2_ could also affect responses to hypoxia, hypercapnia and hypercarbia, impairing the respiratory adjustments to and resuscitation from potentially life‐threatening conditions [29, 33, 34]. Additionally, PGE_2_ could directly inhibit respiration through actions in the brainstem [13, 29, 35]. Thus, high PGE_2_ levels could impair the infant's ability to adequately respond to increased pCO_2_, potentially increasing the risk of life‐threatening collapse when ventilation is hindered.

**TABLE 4:** Plausible causes of SUPC discovered in the investigation following the event.

Cause	*n* (%)		*n* (%)
Unknown	129 (86)		
Heart defect	7 (6)		
		SCN5A	1 (1)
		Patent ductus arteriosus, atrial septal defect	1 (1)
		Atrial septal defect, patent foramen ovale	1 (1)
		Patent ductus arteriosus	3 (3)
		Patent foramen ovale	1 (1)
Early onset GBS sepsis	3 (3)		
Coagulation Disorder	1 (1)		
Meningoencephalitis	1 (1)		
Trisomy 21	1 (1)		
Congenital central hypoventilation syndrome	1 (1)		
Panhypopituitarism	1 (1)		
Thrombocytopenia	1 (1)		
Oesophageal atresia	1 (1)		
Laryngomalacia	1 (1)		
IVH after vacuum extraction	1 (1)		
Metabolic disease	1 (1)		

**FIGURE 4:**
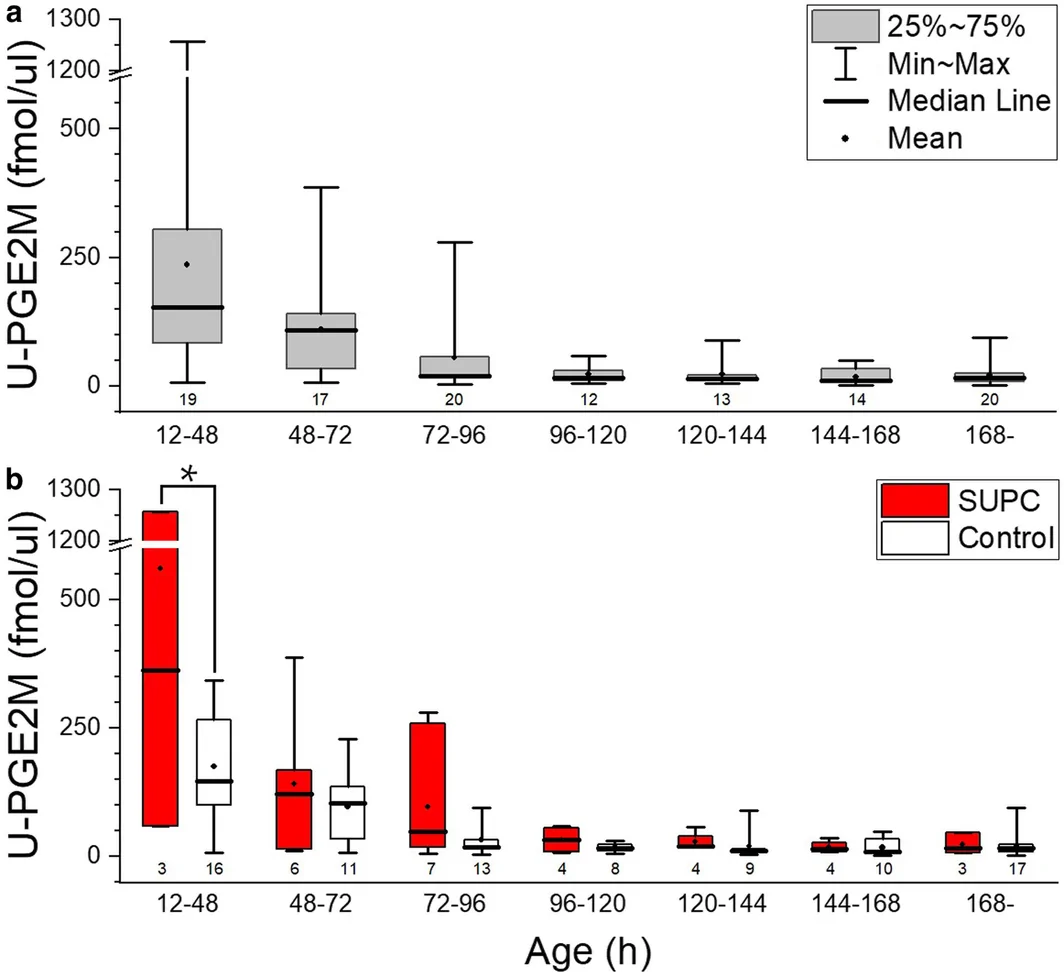
PGE_2_ levels are high immediately after birth. In 10 sudden unexpected collapse (SUPC) cases and 14 age‐matched controls, we found high levels of a prostaglandin E_2_ (PGE_2_) metabolite in urine during the first 3 days (a). The high PGE_2_ levels correlate with the period when most SUPCs occur, with levels in SUPC cases being significantly higher the first 48 h (b). SUPC *n* = 10 patients with 31 samples and age‐matched controls *n* = 14 with 84 samples during the first 7 postnatal days, number of samples/group below the respective boxplots. *p < 0.05, Urinary Prostaglandin E_2_ metabolites (U‐PGE2M).

## 4 Discussion

We have provided novel evidence that all newborn infants are at risk of SUPC in the first 24 h of birth. Moreover, SUPC was associated with early unsupervised skin‐to‐skin care, prone position and lower birth weight. These risk factors also appeared in a previous study regarding SUDI during the first week of life [19]. While the aetiology of SUPC remains poorly understood, we hypothesize a connection to brainstem dysfunction related to autonomic and respiratory control, potentially related to birth‐induced release of PGE_2_. Furthermore, we highlighted some potential risk factors for SUPC. Firstly, the initial hours postpartum were crucial. Awareness of these novel data should lead to extra focus on the interaction between newborn infants and caregivers during the first hours of life. Secondly, our results indicated that lower birth weight and gestational age increased the risk of SUPC. This could potentially be related to these infants being less developed or their gestational age overestimated during pregnancy. Finally, co‐bedding and early unsupervised skin‐to‐skin care emerged as risk‐factors for SUPC.

Skin‐to‐skin care and primiparity as risk factors for SUPC have been suggested in several previous studies [1, 9, 18, 36, 37]. Recently, Colvin et al. showed that bed sharing and parents falling asleep during feeding increased the risk of death following SUPC [19]. We presented that 70% of the SUPC ending in death occurred during skin‐to‐skin care when the parent was asleep. There are, however, benefits with skin‐to‐skin care for the physiological adaptation of the infant [36]. Therefore, this emphasized the importance of correct surveillance and parental information [15, 18, 38] regarding safe skin‐to‐skin care as suggested by the British Association of Perinatal Medicine [15, 39].

The incidence and outcome of early GBS infection has been investigated in a Swedish study [21] with an incidence calculated at 34 per 100 000 irrespective of gestational age. This was higher than our data of 17 per 100 000. Including all infants in our study resulted in an incidence of early GBS at 32 per 100 000. While 10 infants died following SUPC, not a single infant born at term (gestational age > 35 weeks) died due to GBS sepsis. This is likely due to strict guidelines and vigilance with regards to the management of GBS sepsis. Among all infants with early onset GBS sepsis, the mortality was 4.8% [21]. Thus, despite early onset GBS cases having poorer postnatal adaptation (lower APGAR scores), they had a better outcome in terms of mortality compared to SUPC. Notably, compared to SUPC events, early onset GBS sepsis is well known and still considered the leading cause of severe neonatal morbidity [40]. SUPC was almost twice as common as early onset GBS sepsis in term or near‐term infants but does not even have a specific ICD‐10 code.

As nine of the 10 infants who died also underwent forensic post‐mortem, we were able to provide detailed information on the cause of death and clues towards identifying an aetiology of SUPC. Initially, we postulated that unless any obvious pathology was detected at post‐mortem, SUDI cases following SUPC events would be diagnosed as SIDS, due to the similarities in risk factors such as co‐bedding. However, the post‐mortem reports stated that the cause of death was based on both clinical findings at post‐mortem and known circumstances surrounding the death. In this study, the most prevalent cause of death was hypoxic brain and organ injury, and only one case was due to unknown aetiology. Notably, these cases would most likely all have been diagnosed as SIDS five to 10 years ago without adequate death scene investigations, but now show a correlation with accidental asphyxiation. The diagnostic shift away from SIDS occurred late in Sweden seen from an international perspective. In the 1990s and early 2000s, countries such as Australia, New Zealand, and the USA shifted in cause of death from SIDS to other causes such as accidental suffocation or unexplained cause of death when findings at the death scene were taken into account [27]. In most SUDI cases in Sweden, there was still a lack of death scene investigations. However, in SUPC cases, this information was more readily available. Thus, the number of SUPC events preceding SIDS was lower than expected, but conversely, SUPC events might lead to SUDI. Furthermore, SUPC, SUDI and SIDS shared many risk factors such as prone position, primiparity, low infant birth weight, bedsharing and unsupervised early skin‐to‐skin care [16, 18, 19].

## 5 Strenghts and Limitations

This is, to the best of our knowledge, the largest study of SUPC. Apart from a long study period and a large cohort, a strength compared to previous studies [1, 6, 10, 23, 25, 27] is the protocol for identification of the cases. The retrospective design and use of previously recorded electronic health records data did however limit the reliability and extent of data in individual cases. We have actively looked for possible cases by searching for related diagnoses among all children born in the Stockholm region during the study period and thoroughly reviewed electronic health records. This showed that underreporting is an important bias in previous studies that relied on medical staff to report cases to the researchers and registries. It also explains why we identified a higher incidence than expected. A similar method was utilized by [41], where only the relation of SUPC to a single diagnosis, cerebral palsy, was investigated. Furthermore, the Swedish personal identification number system where a personal identification number is given to each individual at birth and follows the individual throughout life has allowed us to retrieve all electronic health record data of the included cases. Thus we have been able to identify risk factors and outcomes more extensively.

## 6 Conclusion

We conclude that SUPC is a potentially catastrophic event more common than initially anticipated and could be prevented by improvement and implementation of existing guidelines on infant care during the first days of life [9, 15, 16, 17, 18, 38], especially for the slightly smaller and earlier born infants. Further characterization of potential risk factors as well as the effects of recently implemented guidelines is needed. To improve comparability between studies, international criteria and specific diagnosis code should be created. Crucially, this study underlines the importance of promoting safe skin‐to‐skin care and sleep within the hospital setting and in the community.

## Author Contributions

**David Forsberg:** writing – original draft, investigation, methodology, validation, writing – review and editing, visualization, formal analysis, funding acquisition. **Louise Steinhoff:** investigation, writing – review and editing, formal analysis, methodology, validation. **Gustaf Drevin:** investigation, writing – original draft, formal analysis. **Helena Idborg:** formal analysis, methodology. **Nicolas J. Pejovic:** validation, writing – review and editing. **Eric Herlenius:** conceptualization, investigation, funding acquisition, methodology, validation, writing – review and editing, project administration, supervision.

## Funding

The present study was supported by grants to Eric Herlenius (EH) from the Swedish Research Council (2019‐01157 and 2023‐02613), the Stockholm County Council (FoUI‐966449), the Swedish National Heart and Lung Foundation (2018‐0505 and 2021‐0579), the Swedish Brain foundation (FO2019‐0087, FO2021‐0313, FO2023‐0231 and FO2025‐0454), Freemasons Children's House foundations and Karolinska Institutet. Dr. David Forsberg (DF) was partly supported by the Freemasons Children's House Foundation Scholarship, the Swedish Infant Death foundation and the Society for Child Welfare. The funders did not participate in the study design or conduct.

## Conflicts of Interest

The authors declare no conflicts of interest.

## Supporting information


**Figure S1:** Post‐SUPC investigations. Use of post‐SUPC investigations and blood sampling presented as percentage of cases per year. Although *R* ^2^ were low, we could observe declining trends in the use of echocardiography and electroencephalography after initial blood gas analysis. However, acute blood sampling and blood gas analysis increased during the study period.

**Table S1:** Reasons for exclusion as the postnatal collapse was not unexpected.

**Table S2:** Parameters identified in medical records.

**Table S3:** Cause of death.

## Data Availability

The data analysed in this study is subject to the following licences/restrictions: Since the datasets are personal health records (PHRs), requests to access these datasets should be directed to eric.herlenius@ki.se.
