# Supplementary material for: Sudden Unexpected Postnatal Collapse: A Retrospective Cohort Study Describing Incidence, Risk Factors and Role of Perinatal Transition
Source: Acta Paediatr. 2026 Jul 13;115(12):2725–34. doi: 10.1111/apa.70670 (PMC13747700; doi:10.1111/apa.70670)
Supplement: Supplementary file 1 [file APA-115-2725-s002.docx]

**Table S1. Reasons for exclusion as the postnatal collapse was not unexpected.**

|  | **n** |  | **n** |
| --- | --- | --- | --- |
| **Other reason for exclusion** | **22** | **Malformations** | **27** |
| Maternal drug exposure (opioids) | 1 | Trisomy 21 | 7 |
| Meningitis | 2 | Coarctation of the aorta | 3 |
| Hydrocephalus | 1 | Diaphragmatic hernia | 2 |
| Infection | 11 | Choanal atresia | 1 |
| Repeated vomiting | 1 | Achondroplasia | 1 |
| Irritability with suspicion of meningitis or asphyxia | 4 | Micrognathia | 1 |
| Subgaleal hematoma | 1 | Transposition of the great arteries | 1 |
| Severe anaemia | 1 | Pierre-Robin Syndrome | 1 |
|  |  | Ichthyosis | 1 |
|  |  | Tracheal malformation | 1 |
|  |  | Laryngomalacia | 1 |
|  |  | Single ventricle defect | 1 |
|  |  | Anal atresia | 1 |
|  |  | Arthrogryposis | 1 |
|  |  | Noonan Syndrome | 1 |
|  |  | Pulmonary Stenosis | 1 |
|  |  | Trisomy 8 | 1 |
|  |  | Neck tumour | 1 |
