# Supplementary material for: Sudden Unexpected Postnatal Collapse: A Retrospective Cohort Study Describing Incidence, Risk Factors and Role of Perinatal Transition
Source: Acta Paediatr. 2026 Jul 13;115(12):2725–34. doi: 10.1111/apa.70670 (PMC13747700; doi:10.1111/apa.70670)
Supplement: Supplementary file 2 [file APA-115-2725-s003.docx]

**Table S2. Parameters identified in medical records.**

| Pregnancy and birth | Event | Discharge and follow up |
| --- | --- | --- |
| Apgar score | Age | Neurological status |
| Parity | Last caretaker | Electroencephalogram |
| Maternal smoking | Last breastfeeding | Magnetic resonance imaging |
| Maternal drugs | Who found the baby | Neuropsychological development |
| Type of delivery | Where the baby was found | Motor development |
| Sex | Co-bedding | Metabolic parameters |
| Birth weight | Co-sleeping | Infectious parameters |
| Gestational age | If parent was asleep |  |
| Umbilical artery pH | Blood pH and base excess |  |
| Umbilical artery base excess | Neurological symptoms |  |
|  | Hypothermia treatment |  |
|  | Hypoglycaemia |  |
|  | Ventilation |  |
|  | Continuous positive airway pressure |  |
|  | Intubation |  |
|  | Brain Ultrasound |  |
|  | Electroencephalogram |  |
|  | ECG |  |
|  | Acute blood samples |  |
