# Supplementary material for: Sudden Unexpected Postnatal Collapse: A Retrospective Cohort Study Describing Incidence, Risk Factors and Role of Perinatal Transition
Source: Acta Paediatr. 2026 Jul 13;115(12):2725–34. doi: 10.1111/apa.70670 (PMC13747700; doi:10.1111/apa.70670)
Supplement: Supplementary file 3 [file APA-115-2725-s004.docx]

**Table S3. Cause of death.**

| Cause of death | n (%) |
| --- | --- |
| Bilateral spontaneous pneumothorax* | 1 (10) |
| SIDS | 1 (10) |
| Positional asphyxia | 1 (10) |
| Hypoxic brain injury due to blocked airway or body positioning | 2 (20) |
| Hypoxic brain injury with multiorgan failure of unknown aetiology | 1 (10) |
| Congenital heart malformations | 2 (20) |
| Severe lung disease | 1 (10) |
| Metabolic disease | 1 (10) |
| **Cause of death specified without forensic post-mortem investigation.* | |
